# A genome-wide and candidate gene association study of preterm birth in Korean pregnant women

**DOI:** 10.1371/journal.pone.0294948

**Published:** 2023-11-29

**Authors:** Young Min Hur, Jae Young Yoo, Young Ah You, Sunwha Park, Soo Min Kim, Gain Lee, Young Ju Kim

**Affiliations:** 1 Department of Obstetrics and Gynecology, College of Medicine, Ewha Medical Research Institute, Ewha Womans University Mokdong Hospital, Seoul, Korea; 2 Division of Biobank, Korea National Institute of Health (KNIH), Korea Disease Control and Prevention Agency (KDCA), Cheongju, Korea; Shaheed Rajaei Hospital: Rajaie Cardiovascular Medical and Research Center, ISLAMIC REPUBLIC OF IRAN

## Abstract

Preterm birth (PTB) refers to delivery before 37 weeks of gestation. Premature neonates exhibit higher neonatal morbidity and mortality rates than term neonates; therefore, it is crucial to predict and prevent PTB. Advancements enable the prediction and prevention of PTB using genetic approaches, especially by investigating its correlation with single nucleotide polymorphisms (SNPs). We aimed to identify impactive and relevant SNPs for the prediction of PTB via whole–genome sequencing analyses of the blood of 31 pregnant women with PTB (n = 13) and term birth (n = 18) who visited the Ewha Womans University Mokdong Hospital from November 1, 2018 to February 29, 2020. A genome-wide association study was performed using PLINK 1.9 software and 256 SNPs were selected and traced through protein-protein interactions. Moreover, a validation study by genotyping was performed on 60 other participants (preterm birth, n = 30; term birth, n = 30) for 25 SNPs related to ion channel binding and receptor complex pathways. Odds ratios were calculated using additive, dominant, and recessive genetic models. The risk of PTB in women with the AG allele of rs2485579 (gene name: *RYR2*) was significantly 4.82-fold increase, and the risk of PTB in women with the AG allele of rs7903957 (gene name: *TBX5*) was significantly 0.25-fold reduce. Our results suggest that rs2485579 (in *RYR2*) can be a genetic marker of PTB, which is considered through the association with abnormal cytoplasmic Ca^2+^ concentration and dysfunctional uterine contraction due to differences of *RYR2* in the sarcoplasmic reticulum.

## Introduction

Preterm birth (PTB) refers to delivery before 37 weeks of gestation, and premature neonates exhibit various neonatal morbidity and mortality rates; therefore, it is crucial to predict and prevent PTB due to its significant impact on neonatal health and healthcare resources [1]. PTB accounts for approximately 4–16% of babies born in 2020 across countries [2], making it a substantial public health concern; however, the basic molecular etiology remains unclear, and only about 30–35% of all PTB cases have a specific known cause [3]. There are direct causes such as spontaneous preterm labor and preterm premature rupture of membranes (PPROM), and indirect factors, such as being underweight or obese, risky age range (≤19 years old or ≥35 years old), vitamin C deficiency, depression, anxiety, and chronic stress [1,4–6]. PTB is a complex syndrome resulting from multiple factors.

PTB is recurrent characterized and has a hereditary component [1,7]. In other words, one of the most important risk factors for PTB is previous experience with PTB and the causes tend to recur [1,8]. Compared to the PTB rate in the general population, the probability of PTB in the next pregnancy increases to 17.2% for women with a history of PTB [9]. Moreover, the probability of PTB in a third pregnancy could increase to 28.4% if the first two pregnancies resulted in PTB [9]. Therefore, it seems reasonable to consider genetic predisposition as an approach for the prediction and prevention of PTB.

The Human Genome Project, which decoded the three billion DNA base pairs comprising the human genome, enabled high-throughput biology using next-generation sequencing (NGS) [10]. Moreover, the bioinformatics approach has contributed to tracking down candidate substances capable of affecting genes involved in metabolic pathways of interest and the expression of those genes [10,11]. In particular, we focused on single nucleotide polymorphisms (SNPs), which can be used to investigate the correlation between individual variants in genotypes and disease phenotypes [10–12]. Of the three billion total bases in the genome, approximately 0.1–0.2% of them (three to six million bases) represent genotypic variation between individuals at specific base locations; these variations are called SNPs, and their frequencies are higher than 1% in the population [10,12]. The polymorphism could be suggested as a mechanism leading to a ‘common complex disease’ that exhibits complex characteristics, such as PTB [10,13,14]. For example, schizophrenia, diabetes mellitus, and autoimmune disorders have already been considered as genomic disorders of ‘common disease-common variant hypotheses’ with various genetic and environmental backgrounds through several studies [10–12].

Therefore, this study aimed to analyze whole blood genomic sequencing in women with PTB and full-term births to explore the related underlying mechanisms and identify potential predictive biomarkers for PTB and targeted treatments.

## Materials and methods

### Study design and participants

This case-control study was conducted as part of the Ewha Preterm Birth Cohort Study, established at Ewha Womans University Mokdong Hospital, Seoul, Republic of Korea, between November 1, 2018 and February 29, 2020. The 31 participants were selected randomly (13 cases of PTB and 18 controls). The study protocol was approved by the Institutional Review Board (IRB) of Ewha Womans University Mokdong Hospital (IRB No. EUMC 2018-07-007). Written informed consent was obtained from all participants at enrollment, and the consent procedure was approved by the IRB. Women with early pregnancy loss, multifetal pregnancy, fetal anomalies, or other obstetric or non-obstetric pregnancy complications were excluded.

PTB was defined as delivery at < 37 weeks of gestation, based on the last menstrual period confirmed or modified by ultrasound evaluation [15]. Spontaneous preterm labor was defined as the presence of intact membranes and regular contractions, while PPROM was defined as the rupture of membranes before the onset of preterm labor [1]. Data on the clinical, demographic, and socioeconomic variables of the participants who had PTB or not were obtained from obstetric and neonatal medical records.

### Blood sample and whole–genome sequencing (WGS) data collection

Blood samples from the subjects were collected in the second or third trimester of gestation. Whole venous blood was collected in a vacutainer tube containing EDTA and centrifuged to allow for the collection of plasma and the buffy coat. The buffy coat enriched with white blood cells was used for DNA extraction. All buffy coats were prepared under optimal conditions within two hours after blood collection and immediately aliquoted and stored at -80°C and not thawed until analyzed.

Genomic DNA was extracted from peripheral blood samples using the Qiagen DNA Blood Mini Kit. DNA quality was assessed using a Nanodrop spectrophotometer and samples with a 260/280 ratio between 1.8 and 2.0 were used for library preparation. Libraries were prepared using the KAPA Hyper Prep Kit and sequenced on an Illumina NovaSeq 6000 platform with 2–150 bp paired-end reads. Quality control of the sequencing reads was performed using FastQC and reads were aligned to the human reference genome (hg38) using the Burrows-Wheeler Aligner. PCR duplicates were marked and removed using Picard Tools, and base recalibration and local realignment around the indels were performed using the GATK software. Variant calling was performed using the GATK 4.3 HaplotypeCaller, and variants were filtered based on read depth, quality, and annotation using the GATK Variant Filtration tool. Functional annotation of the variants was performed using SnpEff 4.3 and VEP along with dbSNP build 154 [16–18]. Quality control measures were taken at each step of the analysis, including checking for outliers and assessing the reproducibility of the results. Based on these procedures, it was possible to obtain 3 GB of genomic data for each sample through sequencing and numbering.

### Genome-wide association study and SNP selection

Using the genomic data obtained from the participants, a genome-wide association study (GWAS) was conducted between cases and controls to evaluate the association between PTB and specific genetic variations. GWAS is the genome-wide scanning of genetic variants in many individuals to identify common variations and genes involved in human diseases [10]. A case-control association test was implemented for PTB, and the results were manually reviewed to rank the sequence variations estimated to correlate with the phenotype. All genetic association analyses were conducted using PLINK 1.9 software, a publicly available genetic association analysis program developed by the Center for Human Genetic Research at Massachusetts General Hospital and the Broad Institute [19]. Individuals with a call rate lower than 95% (more than 5% missing SNP data) and SNPs without data reported in over 5% of previous studies were excluded.

To identify clusters of genes significantly associated with PTB, we performed a pathway analysis of the identified variants using the STRING (Search Tool for the Retrieval of Interacting Genes/Proteins) biological database, which predicts protein-protein interactions [20]. Basic information regarding the genes was obtained from The National Center for Biotechnology Information and retrieved from PubMed, an archive of biotechnology and medical article indices.

### Validation of 25 SNPs related to PTB by genotyping

Among the pathways traced through protein-protein interactions of genes associated with differentially expressed SNPs in PTB, two clusters were selected and compared with pathways reported in the literature. Subsequently, the selected 25 SNPs were validated in another group of 60 pregnant women (30 with PTB and 30 with controls). The SNPs were validated by the Kompetitive allele specific PCR (KASP) in 60 whole blood samples. We used flanking sequences, with the evaluated SNPs indicated in the parentheses, to design the KASP assays. All custom SNP assays were designed by the corresponding company (LGC Genomics; Hoddesdon, Herts, UK). KASP assays were performed in a volume of 5 μL using 384-well plates, and low ROX was used as a passive reference dye. Two probes ensured the allelic specificity of the TaqMan assay: one was labeled with FAM and the other with VIC. In the KASP assays, bi-allelic discrimination was achieved through the competitive binding of two allele-specific forward primers: one labeled with FAM and the other with HEX. These reporter dyes were detected independently using real-time quantitative PCR instruments with excitation sources and emission filters at the respective wavelengths. Genotyping was performed using the QuantStudio 5 Real-Time PCR System (Applied Biosystems, Waltham, MA, USA).

### Statistics

The maternal demographics and clinical characteristics of the term birth and PTB groups were compared using the Student’s t-test for continuous data and the Chi-square test for categorical variables. Maternal age and body mass index (BMI) were not normally distributed; therefore, the median values were compared using the Mann-Whitney U test. A *P*-value < 0.05 was considered statistically significant. In the GWAS, we assessed deviations from the Hardy-Weinberg equilibrium (HWE) within our population for each SNP using the Chi-square test in PLINK 1.9 software. A *P*-value threshold of < 7.64 x 10^−4^ was used to determine statistical significance.

Candidate genes selected by tracing pathways were analyzed using three genetic models (additive, dominant, and recessive) and the logistic regression model analysis. Each model makes different assumptions regarding the genetic effects on phenotypes in the data [21,22]. For a single SNP with two alleles (D = disease-causing allele and N = allele not causing the disease), the dominant model (for the D allele) assumes that having one or more copies of the D allele increases the risk compared to the risk associated with N. Therefore, the genotypes DD or ND have a higher risk. In the recessive model (for the D allele), it is assumed that two copies of the D allele are required to alter the risk. Therefore, individuals with the genotype DD were compared to those with genotypes ND and NN. Finally, the additive model (for the D allele) assumed a linear and uniform increase based on the number of copies of the disease-causing allele (D). Thus, the risk for ND is k, and that for DD is 2k [21–23]. Odds ratios (OR) and their 95% confidence intervals (CI) in relation to the genotypes of the 25 SNPs from the two clusters were calculated following each model after adjustment for covariates. Statistical analyses were performed using the Statistical Package for Social Sciences (SPSS, Version 20 Chicago, NY, USA).

## Results

Table 1 shows the demographic and clinical characteristics of the term birth and PTB groups in the GWAS. The average maternal age in both groups was around 31 to 32 years old. The PTB group was similar to the term birth group in terms of maternal age, pre-pregnancy BMI, nulliparity distribution, and PTB history. A gestational age at delivery was lower for PTB (30.2 weeks, IQR 26.8–33.2) than it was in the controls (39.3 weeks, IQR 38.0–40.0) (*P* < 0.001). As expected, the infant birthweight was 1517.2 ± 580.5 g at PTB and 3241.7 ± 337.3 g at term birth (*P* < 0.001). Apgar score was also significantly different between the two groups, with 1’-6 and 5’-7 (in PTB) vs. 1’-10 and 5’-10 (in term birth) (Table 1). Spontaneous preterm labor and PPROM occurred in eight patients (61.5%) with PTB.

**Table 1:** Clinical characteristics of participants on screening test (n = 31).

Characteristics	PTB (n = 13)	TB (n = 18)	*P*-value
Maternal age (years)	32.0 (29.5–35.0)	31.0 (28.8–34.3)	0.737
Pre-pregnancy BMI (kg/m^2^)	22.3 (20.1–24.3)	21.5 (19.9–25.1)	0.859
Nulliparity (n, %)	9 (69.2%)	12 (66.7%)	0.880
History of prior PTB (n, %)	1 (3.2%)	2 (6.5%)	0.751
GA at birth (weeks)	30.2 (26.8–33.2)	39.3 (38.0–40.0)	<0.001^a^
Birth weight (g)	1517.2±580.5	3241.7±337.3	<0.001 ^a^
Apgar score (1 min)	6.0 (3.5–8.5)	10.0 (8.8–10.0)	0.001 ^a^
Apgar score (5 min)	7.0 (6.0–10.0)	10.0 (10.0–10.0)	0.002 ^a^

Categorical variables are expressed as frequencies (%) and analyzed using the Chi-square test. Continuous variables were expressed as the mean ± standard deviation (SD) by Student’s t-test or median (interquartile range, IQR) using the Mann-Whitney U test.

Abbreviation: PTB, preterm birth; TB, term birth; BMI, body mass index; GA, gestational age.

^a^ Statistical significance was defined as *P* < 0.05.

Table 2 shows demographic and clinical characteristics of term birth and PTB groups in a validation study including a PTB group (n = 30) and a term birth group (n = 30). There were no significant differences between the two groups for age, pre-pregnancy BMI, distribution of nulliparity, and history of preterm birth. On the other hand, the gestational age at birth was 34.3 weeks (IQR 32.0–35.4) at PTB vs. 38.5 weeks (IQR 38.0–39.4) at term birth (*P* < 0.001) and birth weight of the infants was 2100.3±590.3 g at PTB vs. 3145.3±405.0 g at term birth (*P* < 0.001) (Table 2). Apgar score was also significantly different between the two group. In this group, spontaneous preterm labor and PPROM accounted for 24 patients (80%) of PTB cases. As such, the group for GWAS and the validation study group showed similar characteristics.

**Table 2:** Clinical characteristics of participants on validation study (n = 60).

Characteristics	PTB (n = 30)	TB (n = 30)	*P*-value
Maternal age (years)	32.9±4.0	33.5±3.8	0.575
Pre-pregnancy BMI (kg/m^2^)	21.4 (20.0–24.0)	21.3 (19.7–23.5)	0.495
Nulliparity (n, %)	18 (60.0%)	21 (70.0%)	0.489
History of prior PTB (n, %)	2 (6.7%)	2 (6.7%)	1.000
GA at birth (weeks)	34.3 (32.0–35.4)	38.5 (38.0–39.4)	<0.001^a^
Birth weight (g)	2100.3±590.3	3145.3±405.0	<0.001 ^a^
Apgar score (1min)	9.0 (6.0–10.0)	9.0 (8.5–10.0)	0.013 ^a^
Apgar score (5min)	10.0 (8.8–10.0)	10.0 (10.0–10.0)	0.020 ^a^

Categorical variables are expressed as frequencies (%) and analyzed using the Chi-square test. Continuous variables were expressed as the mean ± standard deviation (SD) or median (interquartile range) and were compared using the Man-Whitney test.

Abbreviation: PTB, preterm birth; TB, term birth; BMI, body mass index; GA, gestational age.

^a^ Statistical significance was defined as *P* < 0.05.

### GWAS and SNP selection

**Fig 1:**
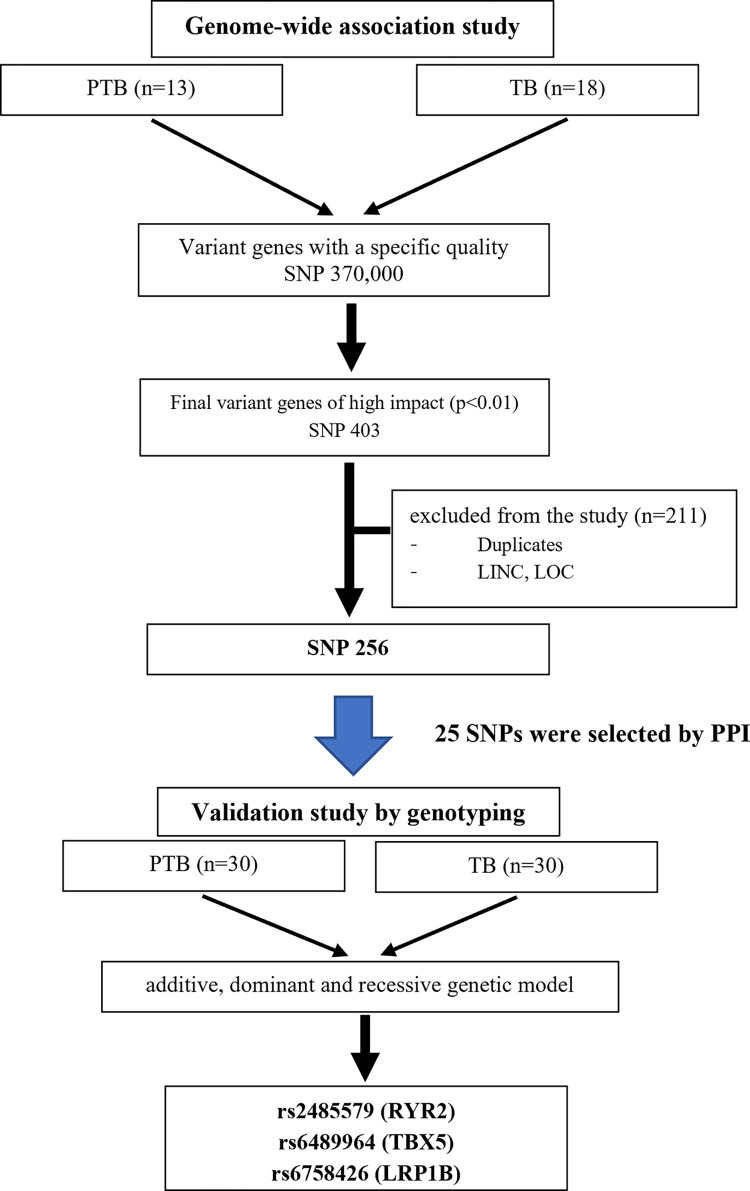
Analytic framework for the identification of PTB related variant genes. The diagram shows the processes used to filter the variants identified via WGS. Abbreviations: PTB, preterm birth; TB, term birth; LINC, long intergenic non-protein coding RNA; LOC in genetics are genes with unknown functions; PPI, protein-protein interactions; WGS, whole genome sequencing.

**Fig 2:**
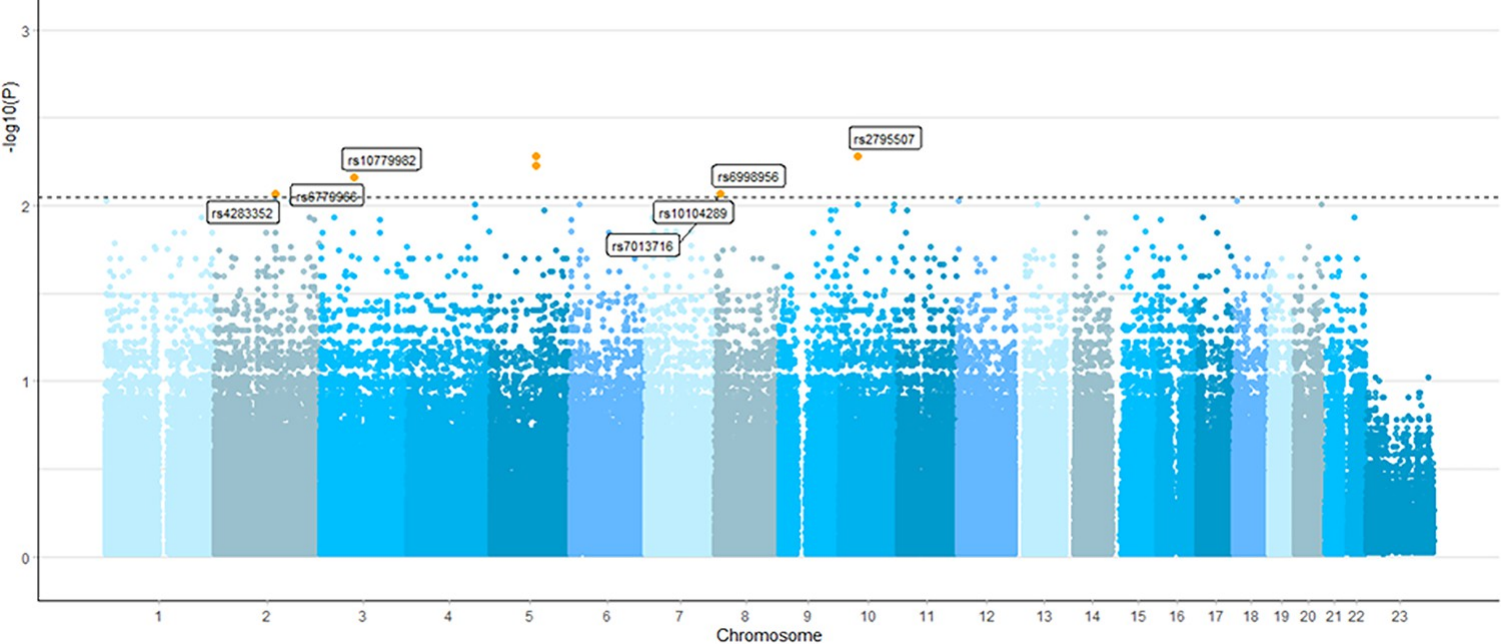
Manhattan plot of GWAS analysis adjusting for PTB. The figure shows a Manhattan plot of all variants across 22 autosomes and X chromosomes, the vertical axis being the -log *P*–value from the statistical test for association. Abbreviations: GWAS, genome-wide association study; PTB, preterm birth.

The analytical framework for identifying of PTB-related variant genes is shown in Fig 1. WGS was performed on blood samples from 31 participants, and the total read number per sample ranged from 20,061,291 to 25,268,416 (median, 22,865,861). Sequence reads from each sample were aligned to the human reference genome from the Ensembl database. Through GWAS, the initial SNPs were 9,355,599 and 667,306 were selected with HWE *P*-value < 7.64 x 10^−4^, and 370,000 candidates were selected by applying a genotype missing rate of 5% and a minor allele threshold of 10%. A total of 403 SNPs were selected based on the high impact level (*P* < 0.01) by further lowering the statistically significant value using the Manhattan plot (Fig 2). Among these, 256 candidate SNPs were selected after excluding overlapping values, long intergenic non-protein coding RNA (LINC), and unknown functional genes (LOC). In some cases, variants were detected at two or more loci (S1 Table).

We examined the connectivity between genes by protein-protein interactions using STRING to explore the role of 256 candidate SNPs in preterm birth. Among several protein networks, a cluster containing *TBX5*, *RYR2*, *AKAP6*, and *RPS6KA2* and a cluster containing *IFNA21*, *LIFR*, *IL21*, *LRP1B*, *NTRK2*, *CSMD1* (including two loci), *FHIT* (including 13 loci), and *GPM6A* were selected. The former is a cluster associated with ion channel binding and junctional sarcoplasmic reticulum (SR) membranes, whereas the latter is a cluster associated with receptor complexes (S2 Table).

### Validation of 25 SNPs related to PTB by genotyping

For the 25 SNPs, the two groups were compared using the KASP assay, and the cases difficult to design primers were analyzed using Sanger sequencing (S3 Table). Nevertheless, the cases with low call rates were not included in these analyses. The results were analyzed using three genetic models (additive, dominant, and recessive), and are shown in Table 3. The frequency of the AG heterozygote allele in rs2485579 (gene name: *RYR2*) was 51.9% in the PTB group and 13.8% in the control group. The risk of PTB in patients with the AG allele was significantly increased as OR 4.82 (dominant model; 95% CI, 1.34–17.32) (*P* = 0.016). In case of rs6489964 (gene name: *TBX5*), the risk of PTB was seemingly reduced with an OR of 0.25 (dominant model; 95% CI, 0.08–0.82) for the AG allele (*P* = 0.022). Lastly, rs6758426 (gene name: *LRP1B*) showed increased risk of PTB of the CT allele with OR 3.50 but it was not significant (dominant model; 95% CI, 0.89–13.75) (*P* = 0.074) (Table 3).

**Table 3:** Association of 25 SNPs with preterm birth risk, OR^a^ value (95% confidence intervals).

Gene name	SNP	PTB		TB		Additive	Dominant	Recessive
		N	%	N	%			
TBX5	rs6489964							
	AA	19	70.4	12	41.4			
	AG	3	11.1	9	31.0		0.25 (0.08–0.82)^b^	
	GG	5	18.5	8	27.6	0.55 (0.27–1.11)		0.70 (0.18–2.73)
RYR2	rs2485579							
	AA	13	48.1	24	82.8			
	AG	14	51.9	4	13.8		4.82 (1.34–17.32)^b^	
	GG	0	0	1	3.4			
AKAP6	rs1950695							
	CC	22	81.5	23	79.3			
	CT	5	18.5	5	17.2		0.82 (0.19–3.50)	
	TT	0	0	1	3.4			
RPS6KA2	rs6909289							
	CC	18	69.2	16	55.2			
	CT	8	30.8	13	44.8		0.45 (0.14–1.43)	
	TT	0	0	0	0			
IFNA21	rs2891157							
	GG	21	77.8	22	75.9			
	GC	6	22.2	7	24.1		0.89 (0.24–3.21)	
	CC	0	0	0	0			
LIFR	rs3097235							
	GG	12	50.0	12	42.9			
	GA	12	50.0	16	57.1		0.82 (0.26–2.55)	
	AA	0	0	0	0			
LRP1B	rs6758426							
	CC	18	66.7	25	86.2			
	CT	9	33.3	4	13.8		3.50 (0.89–13.75)	
	TT	0	0	0	0			
NTRK2	rs531904							
	GG	19	70.4	19	65.5			
	GA	5	18.5	8	27.6		0.91 (0.27–3.10)	
	AA	3	11.1	2	6.9	1.02 (0.43–2.44)		1.42 (0.20–9.89)
CSMD1	rs2189890							
	TT	19	70.4	25	86.2			
	TC	7	25.9	3	10.3		3.05 (0.68–13.67)	
	CC	1	3.7	1	3.4	2.05 (0.63–6.70)		1.17 (0.06–24.40)
	rs2627403							
	TT	21	77.8	19	65.5			
	TC	6	22.2	9	31.0		0.74 (0.20–2.73)	
	CC	0	0	1	3.4			
FHIT	rs2736743							
	AA	25	92.6	25	86.2			
	AG	2	7.4	4	13.8		0.46 (0.60–3.48)	
	GG	0	0	0	0			
	rs2205351							
	TT	25	92.6	25	86.2			
	TC	2	7.4	4	13.8		0.46 (0.60–3.48)	
	CC	0	0	0	0			
	rs2205350							
	TT	24	88.9	25	86.2			
	TG	3	11.1	4	13.8		0.87 (0.15–5.21)	
	GG	0	0	0	0			
	rs2205349							
	CC	25	92.6	25	86.2			
	CA	2	7.4	4	13.8		0.46 (0.60–3.48)	
	AA	0	0	0	0			
	rs2594146							
	TT	25	92.6	25	86.2			
	TA	2	7.4	4	13.8		0.46 (0.60–3.48)	
	AA	0	0	0	0			
	rs2594147							
	TT	24	88.9	25	86.2			
	TC	3	11.1	4	13.8		0.79 (0.13–4.81)	
	CC	0	0	0	0			
	rs2594145							
	AA	25	92.6	25	86.2			
	AG	2	7.4	4	13.8		0.46 (0.60–3.48)	
	GG	0	0	0	0			
	rs2736741							
	AA	25	92.6	25	86.2			
	AG	2	7.4	4	13.8		0.46 (0.60–3.48)	
	GG	0	0	0	0			
	rs2736742							
	CC	25	92.6	25	86.2			
	CT	1	3.7	4	13.8		0.46 (0.06–3.48)	
	TT	1	3.7	0	0			
	rs2594148							
	AA	25	92.6	25	86.2			
	AG	2	7.4	4	13.8		0.46 (0.60–3.48)	
	GG	0	0	0	0			
	rs2594150							
	CC	25	92.6	25	86.2			
	CA	2	7.4	4	13.8		0.46 (0.60–3.48)	
	AA	0	0	0	0			

^a^ OR: odds ratio, adjusted for age, BMI, and history of PTB.

^b^ *P* < 0.05.

Abbreviations: SNPs, single nucleotide polymorphisms; PTB, preterm birth; TB, term birth; BMI, body mass index.

### The association between PTB and rs2485579 of *RYR2*

Table 4 shows the clinically different characteristics between AA homozygous women and AG heterozygous women for rs2485579 in *RYR2*. Only cases of spontaneous preterm labor, excluding PPROM and other medical causes, were considered for demonstrating the association between rs2485579 and dysfunctional uterine contraction. For AA homozygotes, spontaneous preterm labor events during pregnancy were 40.6%, compared to 70% for AG heterozygotes. Among them, cases of PTB (failure to be managed by tocolysis) were 85.7% in AG heterozygotes and 61.5% in AA homozygotes. In other words, women with the AG allele of rs2485579 have a high probability of spontaneous preterm labor during pregnancy and failure to be treated with tocolysis.

**Table 4:** The clinical different features about spontaneous preterm labor between women who have AA homozygote and AG heterozygote of rs2485579.

		sPTL event (-)	sPTL event (+)			Tocolysis	
	Total		Total	Term birth	Preterm birth	Success to management	Fail to management
rs2485579	N	N (%)	N (%)	N (%)	N (%)	N (%)	N (%)
AA	32	19 (59.4)	13 (40.6)	5 (38.5)	8 (61.5)	4 (44.4)	5 (55.6)
AG	10	3 (30)	7 (70)	1 (14.3)	6 (85.7)	1 (25)	3 (75)

Only cases of spontaneous preterm labor, excluding PPROM and other medical causes, were considered.

Abbreviations: sPTL, spontaneous preterm labor; PPROM, preterm premature rupture of membrane.

## Discussion

In this study, the risk of PTB in women with the AG allele of *RYR2* significantly increased, with and odds ratio of 4.82, and decreased in women with the AG allele of *TBX5* with an odds ratio of 0.25. Moreover, the risk of PTB in women with the CT allele of *LRP1B* was increased by 3.5-fold although it was not significant.

Several studies have revealed an association between PTB and specific SNPs and are accumulating to publically-available database. Wang et al. concluded that functional SNPs in the *SERPINH1* (serpin family H member 1) gene were associated with PPROM in African Americans of 244 cases and 358 controls [8]. The *SERPINH1* gene encodes a heat shock protein (Hsp47) localized to the endoplasmic reticulum, which serves as a chaperone that stabilizes the collagen triple helix [8,24]. Therefore, they concluded that this variant may affect amniotic instability [8]. Moreover, rs879293 (an SNP in the tissue plasminogen activator gene) and rs4986791 (an SNP also known as Thr399lle in the *TLR4* (toll-like receptor 4) gene) in maternal DNA are also associated with PTB [25,26]. According to previous studies about the association between a disease and specific SNPs, related candidate genes were selected through publically-available database and grouped based on pathways. And then, it was validated through such as target sequencing using whole blood samples from large groups of participants [8,27–30]. In case of this study, despite the small sample size of this study, the analytic flow seems to be similar to other studies using established PTB-related gene database in terms of pathways such as smooth muscle contraction or signaling pathways [28]. In addition, we calculated the *P*-value considering the false discovery rate and selected the PTB-related SNPs by pathway analysis. Still, future research seems to be needed for larger participants and through comparison with established genetic databases.

In this study, the risk of PTB in women with the AG allele of rs2485579 in *RYR2* was significantly 4.82-fold increase. The *RYR2* is a second isoform of the ryanodine receptor located in the SR membrane of smooth muscles that releases SR Ca^2+^ into the cytoplasm [31–36]. Uterine myometrial cells are smooth muscle cells and their contractility is driven by a transient increase in intracellular calcium levels [31,33–35,37]. The SR in myometrial cells can buffer intracellular Ca^2+^, and therefore the *RYR2* seems to play an important role to maintain Ca^2+^ concentration [37]. If there is dysfunctional intracellular Ca^2+^ concentration in uterine myometrial cells before 37 weeks of gestation, it can present the phenotype of preterm labor. This concept is also important when using tocolytic agents to manage preterm labor. If rs2485579 affects the *RYR2* expression or function, it can change the Ca^2+^ load through the SR, and as a result, the cytoplasmic Ca^2+^ concentration can change, resulting in dysfunctional uterine contraction. Although the association between the SNP and *RYR2* function could not be experimentally proven, the meaningful clinical features according to different alleles of rs2485579 were analyzed in Table 4. According to another study that analyzed the association between specific SNPs and PTB, six SNPs in *KCNN3*(encodes the small conductance calcium-activated potassium channel subfamily N, member 3) were associated with PTB [38]. Although the variations were located within the intronic region and could not experimentally proven, they suggested the influence of the potassium channels within the myometrium as a major mediator of uterine relaxation by association testing [38]. Yet, it still seems necessary to verify the effect of specific SNPs on gene expression or function.

**Fig 3:**
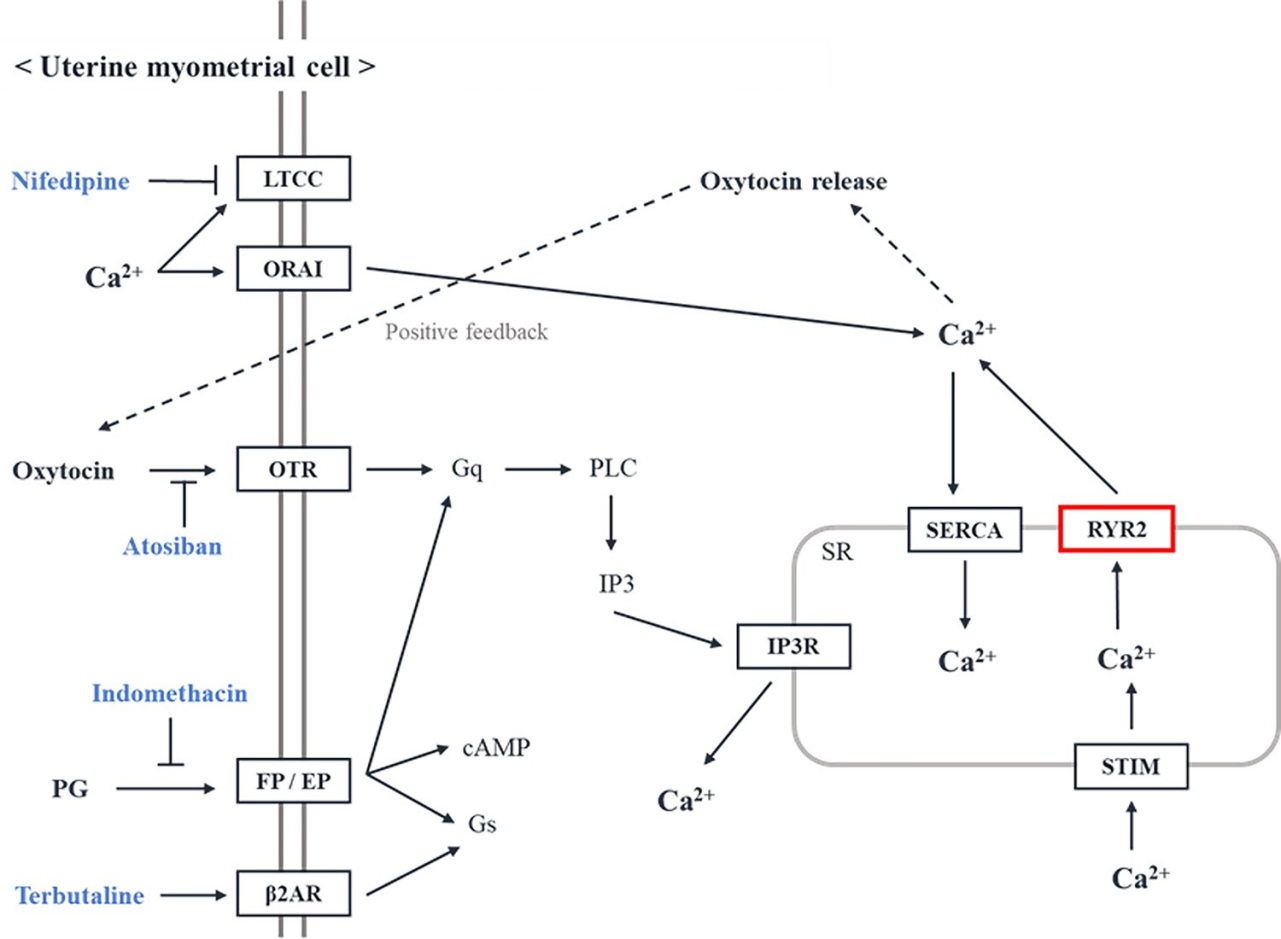
Mechanisms of uterine contractility and function of known tocolytic agents. Ca^2+^ entry into the cell can occur through voltage-gated Ca^2+^ channels and other channel complexes. Ca^2+^ also effluxes from the SR through the *RYR2* and other channels to increase intracellular Ca^2+^ concentration. If a functional problem occurs with *RYR2*, the regulation of intracellular Ca^2+^ concentration will be disrupted and the status will ultimately affect dysfunctional uterine contractions. Abbreviations: LTCC, L-type calcium channel; ORAI, ORAI calcium release-activated calcium modulator; OTR, oxytocin receptor, PG, prostaglandin; EP, prostaglandin E receptor; FP, prostaglandin F receptor; β2AR, β2 adrenergic receptor; PLC, phospholipase C; IP3, inositol 1,4,5-triphosphate; IP3R, IP3 receptor; SR, sarcoplasmic reticulum; SERCA, sarcoplasmic/endoplasmic reticulum calcium ATPase; RYR2, ryanodine receptor 2; STIM, stromal interaction molecule.

The importance of these genes and their pathways in PTB are difficult to speculate. Nevertheless, these results can be plausible and the possible scenario can be considered. The SR in myometrial cells can buffer intracellular Ca^2+^, and if there is problem with *RYR2*, dysfunctional uterine contraction can be shown [37]. Furthermore, it may also show different reactions to tocolytic agents for management of premature uterine contraction. The results of the genomic polymorphism variant analysis described above also can be are applicable to personalized predictive medicine. Several tocolytic agents inhibit molecular mechanisms related to contractions in preterm labor (Fig 3) [1,35,37,39]. These include Ca^2+^ channel blockers, which target L-type calcium channels; COX2 inhibitors, which target prostaglandins; β2 adrenergic receptor agonists, which increase cAMP to cause Ca^2+^ desensitization; and the oxytocin receptor antagonists atosiban [1,37]. However, they do not consider Ca^2+^ shifts through *RYR2* in the SR membrane. Although a Ca^2+^ channel blocker targets L-type calcium channels, the response to tocolysis may be different from patients who have a major allele of the SNPs if there is a functional problem with *RYR2* influencing Ca^2+^ concentration in the cytoplasm. Therefore, the expected response to the same tocolytic agent may be different depending on whether minor allele of G is in rs2485579 in *RYR2*. This finding suggests the possibility of personalized predictive medicine for genetic diversity. It will provide supporting data for determining personalized doses and durations of the same drug for all patients if more data on genetic diversity are accumulated.

On the other hand, the risk of PTB in women with the AG allele of rs7903957 in *TBX5* was significantly 0.25-fold reduce. *TBX5* is a member of the T-box transcription factor family, and is primarily known for its roles in cardiac and forelimb development [40]. Patients with dominant mutations in *TBX5* are characterized by Holt-Oram syndrome, and show defects of the cardiac septum, cardiac conduction system, and the anterior forelimb [40]. Research on specific SNPs in *TBX5* also have been performed and revealed a significant association with congenital heart disease [41]. According to S2 Table, *TBX5* is included in the cluster associated with cardiac left ventricle morphogenesis and in the dominant genetic model analysis, rs6489964 with minor alleles reduced the risk of PTB. Although it is difficult to explain the relationship between *TBX5* and PTB, based on our results, we can propose several hypotheses to describe the decrease in PTB risk associated with the AG allele of *TBX5*. In some cases, tocolytic agents cause side effects such as tachycardia or dyspnea associated with tocolytic agents in pregnant women [1,42]. If the side effects are severe and uncontrolled, the medication should be discontinued, and the risk of PTB increases owing to failure of management. However, if the above series of adverse events on the cardiovascular system in women with a specific minor allele of functional SNPs in *TBX5* do not occur, tocolytic agents can be used only to prevent PTB, thereby decreasing the risk of PTB. However, further studies are required to confirm these hypotheses.

In Addition, rs6758426 in *LRP1B* showed increased risk of PTB of the CT allele but it was not significant. The *LRP1B* gene encodes a member of the low-density lipoprotein (LDL) receptor family [43]. According to S2 Table, *LRP1B* is included in the JAK-STAT signaling pathway and cytokine-cytokine receptor interaction. In general, infection induces uterine contraction, rupture of membrane, cervical softening by promoting changes in prostaglandin, matrix metalloproteinases, or oxytocin receptors through cytokines secretion and the cytokine like IL-6 activate the JAK/STAT3 signaling pathway [44]. Moreover, disruption of this gene has been reported in several types of cancer, and the *LRP1B* protein is also found at the cell surface receptor involved in receptor-mediated endocytosis in the fetal brain [43,45,46]. *LRP1B* was revealed to suppress the activation of IL-6-JAK-STAT3 by several cancer-related previous studies [43,45,47,48]. Similarly, this pathway also can be applied to the situation of PTB. According to previous studies, the association between IL-6 and PTB has been already revealed [49]. The IL-6-JAK-STAT3 induces inflammation reactions and it can cause PTB consequently. If there was a problem with the LRP1B role due to a minor allele of rs6758426, IL-6-JAK-STAT3 activation would not be suppressed. Consequently, the risk of PTB would be higher than a control group. In addition, we can consider that LRP1B is related to the sensitivity to stress affecting to PTB [50–52]. Stress can cause spontaneous preterm labor, and one potential mechanism for stress-induced PTB is the premature activation of the placental-adrenal endocrine axis [1,52]. Elevation of cortisol due to maternal psychological stress increases adult and fetal adrenal steroid hormone production, leading to early loss of uterine quiescence [1,52].

The strength of this study was that specific SNPs related to PTB were identified through experimental verification through GWAS from direct DNA extraction and WGS by collecting whole blood samples from participants for finding novel SNPs. However, this study had several limitations. First, the statistical power may be low owing to the small sample size. This is because we chose the experimental method of analyzing WGS directly from blood samples, not using established public data. Moreover, the results were mainly from the dominant model rather than other models owing to the small sample size. Second, no direct evidence of the corresponding SNPs and phenotypes was determined using experimental methods. Instead, several studies have reported results from known gene-gene and gene-environment interactions without experimental proof [38]. Nevertheless, basic comparison experiments, such as functional assays of the selected candidate genes, should be conducted in future studies.

## Conclusions

The association between rs2485579 (in *RYR2*) and dysfunctional uterine contractions suggests a potential role for Ca2+ channels in the SR in PTB. This SNP may serve as a promising genetic marker for PTB prediction and further research in this area. Although this was an exploratory study of the genetic factors of PTB, it is meaningful in that we suggested a candidate potential genetic marker for PTB prediction.

## Supporting information

S1 Table Candidate list of SNPs related to PTB by genome wide association study (n = 256). (DOCX)

S2 Table Reference pathways related to two clusters according to protein-protein interaction. (DOCX)

S3 Table The 25 SNPs and their respective probe sequences of the Kompetitive allele-specific polymerase chain reaction (KASP) genotyping platforms. (DOCX)
