# Supplementary material for: A genome-wide and candidate gene association study of preterm birth in Korean pregnant women
Source: PLoS One. 2023 Nov 29;18(11):e0294948. doi: 10.1371/journal.pone.0294948 (PMC10686439; doi:10.1371/journal.pone.0294948)
Supplement: S1 Table — (DOCX) [file pone.0294948.s001.docx]

**S1 Table.** **Candidate list of SNPs related to PTB by genome wide association study (n=256)**

| **Gene symbol** | **Gene name** | **SNP** | **Alleles** | **MAF** | **P-value** |
| --- | --- | --- | --- | --- | --- |
| ACER1 | Alkaline ceramidase 1 | rs6510889 | T > A, C | 0.36 | 0.005755 |
| ACSBG2 | Acyl-CoA synthetase bubblegum family member 2 | rs1975157 | A > C, G, T | 0.38 | 0.00677 |
| ADAMTS9 | ADAM metallopeptidase with thrombospondin type 1 motif 9 | rs11429228 | - > C | 0.18 | 0.007568 |
| AIG1 | Androgen induced 1 | rs223605 | G > A, C, T | 0.22 | 0.00678 |
| AJAP1 | Adherens junctions associated protein 1 | rs707588 | G > A | 0.36 | 0.007731 |
| AKAP6 | A-kinase anchoring protein 6 | rs1950695 | T > A, C, G | 0.25 | 0.007568 |
| ANKRD35 | Ankyrin repeat domain 35 | rs61816162 | C > G, T | 0.13 | 0.007731 |
| AP5M1 | Adaptor related protein complex 5 subunit mu 1 | rs8004627 | C > G, T | 0.27 | 0.007568 |
| ARHGEF28 | Rho guanine nucleotide exchange factor 28 | rs443295 | A > C, G | 0.03 | 0.007888 |
| ARL8B | ADP ribosylation factor like GTPase 8B | rs3864057 | A > C, T | 0.35 | 0.002962 |
| BABAM2 | BRISC and BRCA1 A complex member 2 | rs35338642 | TT > -, T, TTT, TTTT | 0.10 | 0.007568 |
| BMS1P20 | BMS1 pseudogene 20 | rs3753078 | A > G, T | 0.30 | 0.007568 |
|  |  | rs736898 | T > C, G | 0.24 | 0.007568 |
|  |  | rs9623381 | A > G | 0.21 | 0.007568 |
|  |  | rs12169829 | G > T | 0.21 | 0.007568 |
|  |  | rs35939211 | G > A | 0.46 | 0.005367 |
|  |  | rs36084829 | T > A, G | 0.19 | 0.004105 |
|  |  | rs73880637 | T > C, G | 0.20 | 0.003151 |
|  |  | rs73880635 | C > A, G | 0.22 | 0.003151 |
|  |  | rs117610294 | C > A, G, T | 0.20 | 0.003151 |
|  |  | rs17578409 | C > T | 0.20 | 0.003151 |
|  |  | rs79612806 | G > A, T | 0.19 | 0.003151 |
|  |  | rs74549497 | G > A, C | 0.19 | 0.003151 |
|  |  | rs34503659 | C > A | 0.41 | 0.003151 |
|  |  | rs532708314 | A > C, G | - | 0.003151 |
|  |  | rs75417423 | A > C, T | 0.36 | 0.003151 |
|  |  | rs560536279 | G > A | - | 0.003151 |
|  |  | rs73153802 | C > T | 0.36 | 0.003151 |
|  |  | rs118041409 | A > C, G | 0.20 | 0.003151 |
|  |  | rs35779014 | T > C | 0.19 | 0.003151 |
|  |  | rs2051500 | T > C, G | 0.19 | 0.003151 |
|  |  | rs73153800 | C > T | 0.19 | 0.003151 |
|  |  | rs17578276 | G > C | 0.19 | 0.003151 |
|  |  | rs62224987 | A > C | 0.20 | 0.003151 |
|  |  | rs62224970 | C > T | 0.19 | 0.003151 |
|  |  | rs17493443 | T > A, G | 0.16 | 0.003151 |
|  |  | rs35572677 | C > T | - | 0.003151 |
|  |  | rs115740254 | C > T | 0.20 | 0.003151 |
|  |  | rs9623370 | T > C | 0.20 | 0.003151 |
|  |  | rs116843465 | G > A | 0.20 | 0.003151 |
|  |  | rs62224971 | G > A, T | 0.20 | 0.003151 |
|  |  | rs1034362 | C > A, G, T | 0.20 | 0.003151 |
|  |  | rs2051497 | G > C, T | 0.18 | 0.003151 |
|  |  | rs76446997 | C > T | 0.21 | 0.003151 |
|  |  | rs62224972 | A > C | 0.19 | 0.003151 |
|  |  | rs148013584 | T > C, G | 0.20 | 0.003151 |
|  |  | rs768491575 | A > C, T | 0.19 | 0.003151 |
|  |  | rs62224968 | C > T | 0.15 | 0.003151 |
|  |  | rs755876353 | T > A, C | 0.27 | 0.003151 |
|  |  | rs2051498 | C > T | 0.18 | 0.003151 |
|  |  | rs2051499 | G > A, C, T | 0.18 | 0.003151 |
|  |  | rs748013325 | G > A, C, T | 0.28 | 0.003151 |
|  |  | rs34583798 | C > G | 0.41 | 0.003151 |
|  |  | rs117042445 | A > T | 0.34 | 0.003151 |
|  |  | rs34334205 | C > T | 0.20 | 0.003151 |
|  |  | rs9623377 | T > A, G | 0.20 | 0.003151 |
|  |  | rs201334749 | G > A, C | 0.38 | 0.003151 |
|  |  | rs9623378 | C > T | 0.20 | 0.003151 |
| BTBD3 | BTB domain containing 3 | rs6131311 | G > A | 0.20 | 0.002777 |
| C4BPA | Complement component 4 binding protein alpha | rs2012296 | G > A, T | 0.07 | 0.007731 |
| C8orf87 | Chromosome 8 open reading frame 87 | rs13257578 | C > A, G, T | 0.29 | 0.007731 |
| CASC2 | Cancer susceptibility candidate 2 | rs7073451 | A > G | 0.37 | 0.005825 |
| CASC6 | Cancer susceptibility 6 | rs9363006 | C > T | 0.49 | 0.006228 |
|  |  | rs2485826 | G > A, C | 0.40 | 0.004434 |
|  |  | rs56654412 | T > A, G | 0.48 | 0.004434 |
| CCDC141 | Coiled-coil domain containing 141 | rs397754737 | - | - | 0.006067 |
| CCT6P3 | Chaperonin containing TCP1 subunit 6 pseudogene 3 | rs61232032 | T > - | 0.04 | 0.006067 |
| CDK17 | Cyclin dependent kinase 17 | rs10777785 | C > A, T | 0.35 | 0.007731 |
| CEP120 | Centrosomal protein 120 | rs11241695 | A > G, T | 0.23 | 0.003121 |
| CHN2 | Chimerin 2 | rs60592356 | G > C, T | 0.33 | 0.0006085 |
| CHRNA9 | Cholinergic receptor nicotinic alpha 9 subunit | rs11732518 | T > A, C, G | 0.39 | 0.005755 |
| CIDEA | Cell death inducing DFFA like effector a | rs4797663 | C > G, T | 0.22 | 0.00677 |
| CLSTN2 | Calsyntenin 2 | rs11359814 | TT > -, T, TTT | 0.25 | 0.003151 |
| CMIP | c-Maf inducing protein | rs4243206 | A > C, G, T | 0.23 | 0.005134 |
| COL21A1 | Collagen type XXI alpha 1 chain | rs9396170 | A > G | 0.46 | 0.007219 |
| COX16 | Cytochrome c oxidase assembly factor COX 16 | rs773090825 | ATACAACACC  CTATAA > - | 0.19 | 0.006067 |
|  |  | rs4899344 | T > C, G | 0.11 | 0.006067 |
| CPM | Carboxypeptidase M | rs11177502 | T > A, C, G | 0.15 | 0.00546 |
| CPZ | Carboxypeptidase Z | rs146244782 | A > G, T | 0.16 | 0.007888 |
| CRAT37 | Cervical cancer-associated transcript 37 | rs7168584 | G > A, C, T | 0.18 | 0.007888 |
| CSMD1 | CUB and Sushi multiple domains 1 | rs2189890 | T > C | 0.43 | 0.004231 |
|  |  | rs2627403 | T > A, C | 0.21 | 0.002423 |
| DAOA | D-amino acid oxidase activator | rs9558590 | C > A, T | 0.49 | 0.007731 |
| DCST1 | DC-STAMP domain containing 1 | rs11264304 | T > A, C, G | 0.31 | 0.007731 |
| DIPK1C | Divergent protein kinase domain 1C | rs1559806 | G > A, C, T | 0.50 | 0.0009359 |
| DLGAP1 | DLG associated protein 1 | rs610721 | C > T | 0.17 | 0.004231 |
| DNAH5 | Dynein axonemal heavy chain 5 | rs1823042 | C > A, T | 0.22 | 0.005134 |
| EBF2 | EBF transcription factor 2 | rs144372117 | A > C, T | - | 0.006067 |
| EPHA5 | EPH receptor A5 | rs2882315 | A > G, T | 0.23 | 0.004368 |
| ETNK1 | Ethanolamine kinase 1 | rs3913136 | G > C, T | 0.21 | 0.005134 |
| EXD2 | Exonuclease 3’-5’ domain containing 2 | rs10716345 | TTTT > -, TT | 0.23 | 0.006228 |
| EYA4 | EYA transcriptional coactivator and phosphatase 4 | rs1932747 | T > A, G | 0.18 | 0.003151 |
| FAM149A | Family with sequence similarity 149 member A | rs56129279 | TT > -, T, TTT | 0.12 | 0.007731 |
| FAM162A | Family with sequence similarity 162 member A | rs4306808 | G > A, C, T | 0.17 | 0.007568 |
| FBP2 | Fructose-bisphosphatase 2 | rs616451 | T > A | 0.28 | 0.004263 |
| FGF14 | Fibroblast growth factor 14 | rs536663 | G > C | 0.21 | 0.003121 |
| FHIT | Fragile histidine triad diadenosine triphosphatase | rs2736743 | A > G | 0.17 | 0.006067 |
|  |  | rs2205351 | T > A, C, G | 0.17 | 0.006067 |
|  |  | rs2205350 | T > G | 0.17 | 0.006067 |
|  |  | rs2205349 | C > A, G | 0.17 | 0.006067 |
|  |  | rs2594146 | T > A, C, G | 0.17 | 0.006067 |
|  |  | rs1018374710 | C > T | 0.18 | 0.006067 |
|  |  | rs2594147 | T > A, C | 0.17 | 0.006067 |
|  |  | rs2594145 | A > G, T | 0.17 | 0.006067 |
|  |  | rs2736741 | A > G, T | 0.17 | 0.006067 |
|  |  | rs6793486 | A > G, T | 0.17 | 0.006067 |
|  |  | rs2736742 | C > T | 0.17 | 0.006067 |
|  |  | rs2594148 | A > G, T | 0.17 | 0.006067 |
|  |  | rs2594150 | C > A, T | 0.17 | 0.006067 |
| FLI1 | Fli-1 proto-oncogene, ETS transcription factor | rs593616 | A > G | 0.21 | 0.006067 |
| FOXB1 | Forkhead box B1 | rs4544191 | T > C | 0.22 | 0.0009483 |
| FOXO6 | Forkhead box O6 | rs7539614 | T > A, C, G | 0.43 | 0.00133 |
| FSTL4 | Follistatin like 4 | rs4958262 | T > A, C, G | 0.24 | 0.006102 |
| FSTL5 | Follistatin like 5 | rs2320330 | T > C, G | 0.25 | 0.002485 |
| GLCCI1 | Glucocorticoid induced 1 | rs12667971 | G > A, C | 0.48 | 0.003829 |
| GNAQ | G protein subunit alpha q | rs10781463 | T > A, C, G | 0.18 | 0.00546 |
| GOLGA8B | Golgin A8 family member B | rs397802905 | - | - | 0.002485 |
| GOT2 | Glutamic-oxaloacetic transaminase 2 | rs7205329 | C > T | 0.25 | 0.007731 |
| GPC6 | Glypican 6 | rs7988063 | G > A, C, T | 0.04 | 0.006067 |
|  |  | rs7982384 | A > C, G, T | 0.17 | 0.003221 |
| GPM6A | Glycoprotein M6A | rs7679873 | A > G, T | 0.27 | 0.007731 |
| GPR139 | G protein-coupled receptor 139 | rs8054898 | T > A, C | 0.20 | 0.004368 |
| GPR26 | G protein-coupled receptor 26 | rs10751742 | C > A, G, T | 0.44 | 0.006067 |
| HJV | Hemojuvelin BMP co-receptor | rs16827043 | C > T | 0.14 | 0.007731 |
|  |  | rs75625937 | T > A, C | 0.12 | 0.007731 |
|  |  | rs1830824 | A > C, G, T | 0.14 | 0.007731 |
|  |  | rs79952022 | A > G, T | 0.14 | 0.007731 |
|  |  | rs115674774 | C > A, G | 0.12 | 0.007731 |
|  |  | rs76943208 | T > C | 0.12 | 0.007731 |
|  |  | rs114103440 | T > A, C, G | 0.08 | 0.005134 |
| HPSE2 | Heparanase 2 | rs10591457 | ATATAT > - | - | 0.006067 |
|  |  | rs10786517 | T > A, C, G | 0.16 | 0.006067 |
| HSPB8 | Heat shock protein family B (small) member 8 | rs2727719 | G >A, C | 0.42 | 0.005825 |
|  |  | rs2727672 | T > A, C, G | 0.23 | 0.005825 |
| IFNA21 | Interferon alpha 21 | rs2891157 | G > A, C | 0.32 | 0.002423 |
| IGFBP3 | Insulin like growth factor binding protein 3 | rs4645488 | C > T | 0.16 | 0.004678 |
| IKBKE | Inhibitor of nuclear factor kappa B kinase subunit epsilon | rs2297546 | C > G | 0.36 | 0.006414 |
| IL21 | Interleukin 21 | rs309392 | G > A, C, T | 0.16 | 0.006067 |
| ITGBL1 | Integrin subunit beta like 1 | rs1414307 | A > C | 0.41 | 0.006404 |
| KCNA6 | Potassium voltage-gated channel subfamily A member 6 | rs2109424 | A > C, G, T | 0.13 | 0.007568 |
| KCNJ6 | Potassium inwardly rectifying channel subfamily J member 6 | rs858027 | A > G, T | 0.16 | 0.006121 |
| KCNK13 | Potassium two pore domain channel subfamily K member 13 | rs2151752 | A > G | 0.35 | 0.007568 |
| KCNMB2 | Potassium calcium-activated channel subfamily M regulatory beta subunit 2 | rs11302457 | AA > A, G | 0.17 | 0.007924 |
| KCNQ3 | Potassium voltage-gated channel subfamily Q member 3 | rs6471053 | C > A, G, T | 0.32 | 0.006067 |
| KCTD2 | Potassium channel tetramerization domain containing 2 | rs8078133 | G > A, C | 0.41 | 0.007924 |
| KLF3 | KLF transcription factor 3 | rs6847640 | C > T | 0.35 | 0.007568 |
| LGALS13 | Galectin 13 | rs7246392 | A > C, G, T | 0.18 | 0.007888 |
| LHFPL3 | LHFPL tetraspan subfamily member 3 | rs11763758 | T > A, C, G | 0.24 | 0.002019 |
| LIFR | LIF receptor subunit alpha | rs3097235 | A > C, G, T | 0.47 | 0.002485 |
| LMO3 | LIM domain only 3 | rs35352991 | T > -, TT, TTT | 0.41 | 0.006067 |
| LRP1B | LDL receptor related protein 1B | rs6758426 | T > A, C, G | 0.46 | 0.006067 |
| LRRK1 | Leucine rich repeat kinase 1 | rs147889569 | - > AG, TG | - | 0.005134 |
| MCTP2 | Multiple C2 and transmembrane domain-containing protein 2 | rs12439652 | A > C, G, T | 0.08 | 0.007888 |
|  |  | rs7181323 | C > G, T | 0.48 | 0.005825 |
| MICALCL | MICAL C-terminal like | rs56284182 | TTTTAA > - | 0.29 | 0.007731 |
| MPP7 | MAGUK p55 scaffold protein 7 | rs7903957 | A > C, G, T | 0.09 | 0.006067 |
| MPPED1 | Metallophosphoesterase domain containing 1 | rs4501036 | C > A, T | 0.34 | 0.006102 |
| NAA30 | N-alpha-acetyltransferase 30, NatC catalytic subunit | rs11620752 | T > A, C, G | 0.47 | 0.005367 |
| NBPF26 | NBPF member 26 | rs7540536 | C > G, T | 0.39 | 0.007888 |
|  |  | rs7367041 | G > T | 0.47 | 0.004678 |
| NELL2 | Neural EGFL like 2 | rs1921551 | T > A, C, G | 0.24 | 0.006067 |
| NFILZ | NFIL3 like basic leucine zipper | rs111326801 | T > A, G | 0.06 | 0.003151 |
| NME8 | NME/NM23 family member 8 | rs35391392 | - > AA | 0.47 | 0.005755 |
| NPHP3 | Nephrocystin 3 | rs113651845 | AAA > -, AA | 0.07 | 0.003389 |
| NRIP1 | Nuclear receptor interacting protein 1 | rs1003427 | G > A, C, T | 0.31 | 0.003151 |
| NTRK2 | Neutrophic receptor tyrosine kinase 2 | rs531904 | G > A | 0.30 | 0.004105 |
| OBI1 | ORC ubiquitin ligase 1 | rs112641687 | TT > -, TTT | 0.31 | 0.007924 |
| ODF1 | Outer dense fiber of sperm tails 1 | rs11333932 | AA > -, A, AAA | 0.10 | 0.003341 |
| OTOF | Otoferlin | rs6712188 | G > A, T | 0.49 | 0.006228 |
| OTOR | Otoraplin | rs6075106 | G > A | 0.14 | 0.007731 |
|  |  | rs2876435 | C > A, T | 0.13 | 0.005134 |
| PA2G4 | Proliferation-associated 2G4 | rs34899902 | - > AT | - | 0.00677 |
| PAFAH1B1 | Platelet activating factor acetylhydrolase 1b regulatory subunit 1 | rs10712058 | AA > -, A, AAA | 0.16 | 0.005134 |
| PAMR1 | Peptidase domain containing associated with muscle regeneration 1 | rs1173906 | C > G | 0.17 | 0.006067 |
| PARD3B | Par-3 family cell polarity regulator beta | rs17450742 | C > A, G, T | 0.37 | 0.00677 |
| PAWR | Pro-apoptotic WT1 regulator | rs7963513 | T > A, G | 0.10 | 0.007731 |
| PDCD6IP | Programmed cell death 6 interacting protein | rs4678460 | C > A, G, T | 0.34 | 0.005825 |
| PDPN | Podoplanin | rs12728383 | G > A, C, T | 0.15 | 0.005134 |
| PEMT | Phosphatidylethanolamine N-methyltransferase | rs12951403 | A > C, G, T | 0.03 | 0.005134 |
| PFDN4 | Prefoldin subunit 4 | rs6091876 | C > A, G, T | 0.41 | 0.007924 |
| PGR | Progesterone receptor | rs545835 | A > C, G, T | 0.49 | 0.007731 |
| PIGG | Phosphatidylinositol glycan anchor biosynthesis class G | rs12512697 | C > G, T | 0.12 | 0.003151 |
| PKIB | cAMP-dependent protein kinase inhibitor beta | rs9482277 | C > G | 0.39 | 0.003829 |
| PLD5 | Phospholipase D family member 5 | rs113984735 | GAGGGAGG > -, GAGG | 0.28 | 0.007731 |
| POTEG | POTE ankyrin domain family member G | rs4069111 | T > C, G | 0.48 | 0.006067 |
| PPP1R12A | Protein phosphatase 1 regulatory subunit 12A | rs2698262 | G > A, C | 0.29 | 0.007354 |
| PRRC2B | Proline rich coiled-coil 2B | rs34941974 | - > T | 0.46 | 0.007731 |
| PSAT1 | Phosphoserine aminotransferase 1 | rs3009699 | G > A, C, T | 0.12 | 0.006926 |
| PURPL | P53 upregulated regulator of p53 levels | rs6875557 | C > T | 0.43 | 0.00678 |
| QKI | QKI, KH domain containing RNA binding | rs5881536 | TT > -, T, TTT | 0.17 | 0.005134 |
| RBMS3 | RNA binding motif single stranded interacting protein 3 | rs9834473 | T > A, C, G | 0.32 | 0.002343 |
| RFX8 | Regulatory factor X8 | rs6543084 | T > A, C, G | 0.23 | 0.005367 |
| RIC3 | RIC3 acetylcholine receptor chaperone | rs11041779 | C > T | 0.15 | 0.001747 |
| RIMS1 | Regulating synaptic membrane exocytosis 1 | rs2496540 | C > A, G, T | 0.42 | 0.007888 |
| RPS6KA2 | Ribosomal protein S6 kinase A2 | rs6909289 | T > A, C | 0.26 | 0.005134 |
| RPUSD2 | RNA pseudouridine synthase domain containing 2 | rs569308564 | - > G | - | 0.004392 |
| RUFY1 | RUN and FYVE domain containing 1 | rs12658664 | C > T | 0.49 | 0.006136 |
| RYR2 | Ryanodine receptor 2 | rs2485579 | A > G, T | 0.39 | 0.005134 |
| SDC1 | Syndecan 1 | rs2881927 | T > C | 0.13 | 0.006067 |
| SDK1 | Sidekick cell adhesion molecule 1 | rs10224716 | A > G, T | 0.01 | 0.006067 |
| SERPINB5 | Serpin family B member 5 | rs1509476 | G > A, C, T | 0.23 | 0.004462 |
| SFMBT2 | Scm like with four mbt domains 2 | rs1969825 | G > A, C | 0.22 | 0.004335 |
| SFRP1 | Secreted frizzled related protein 1 | rs973441 | C > A, G, T | 0.45 | 0.007731 |
| SFRP2 | Secreted frizzled related protein 2 | rs796293083 | ATTATTAGTA  ATTATTAGTA  ATTA > - | 0.42 | 0.004392 |
|  |  | rs4283643 | A > G | 0.39 | 0.00536 |
|  |  | rs1878449 | A > G, T | 0.42 | 0.004392 |
|  |  | rs201173203 | - > GG, GGC | 0.42 | 0.004392 |
| SGMS1 | Sphingomyelin synthase 1 | rs7070574 | C > T | 0.32 | 0.007728 |
| SHISA9 | Shisa family member 9 | rs7195543 | T > A, C, G | 0.45 | 0.007568 |
| SHISAL2A | Shisa like 2A | rs11205989 | G > A | 0.46 | 0.007731 |
| SIRPA | Signal regulatory protein alpha | rs2267916 | T > C | 0.28 | 0.00133 |
| SLC12A1 | Solute carrier family 12 member 1 | rs2413889 | C > T | 0.20 | 0.006136 |
| SLC13A5 | Solute carrier family 13 member 5 | rs183379 | C > A, G, T | 0.32 | 0.002485 |
| SLC15A5 | Solute carrier family 15 member 5 | rs3029441 | ATTATT > - | 0.05 | 0.004231 |
| SLC2A3 | Solute carrier family 2 member 3 | rs58279638 | C > A | 0.29 | 0.005825 |
| SMYD3 | SET and MYND domain containing 3 | rs7529566 | A > C, G, T | 0.43 | 0.007568 |
|  |  | rs2069206 | A > C, G, T | 0.28 | 0.00177 |
| SNX16 | Sorting nexin 16 | rs34186034 | TTTT > -, T, TT | - | 0.003389 |
| SNX19 | Sorting nexin 19 | rs6590569 | G > A, C, T | 0.05 | 0.006067 |
|  |  | rs2513526 | G > A | 0.20 | 0.004434 |
| SORBS2 | Sorbin and SH3 domain containing 2 | rs34261449 | A > - | 0.17 | 0.006067 |
| SOX5 | SRY-box transcription factor 5 | rs4963751 | G > A, C, T | 0.18 | 0.006067 |
| SPATA48 | Sperm microtubule inner protein 7 | rs1456906 | G > A, C, T | 0.47 | 0.006102 |
| SPATA8 | Spermatogenesis associated 8 | rs4246289 | C > A, G, T | 0.16 | 0.006067 |
|  |  | rs4965158 | T > A, G | 0.39 | 0.0003622 |
| SPOCK3 | SPARC (osteonectin), cwcv and kazal like domains proteoglycan 3 | rs28754440 | C > G | 0.40 | 0.004678 |
| SPRED2 | Sprouty related EVH1 domain containing 2 | rs397984866 | - | - | 0.002777 |
| SPRY4 | Sprouty RTK signaling antagonist 4 | rs153423 | A > C, G, T | 0.37 | 0.006136 |
| ST8SIA4 | ST8 alpha-N-acetyl-neuraminide alpha-2,8-sialyltransferase 4 | rs409475 | C > G, T | 0.18 | 0.002777 |
| STAB2 | Stabilin 2 | rs4981022 | G > A, T | 0.25 | 0.002375 |
| STXBP6 | Syntaxin binding protein 6 | rs2754076 | G > A, C | 0.24 | 0.006034 |
| SYNJ2BP | Synaptojanin 2 binding protein | rs2332366 | A > C, G, T | 0.13 | 0.002076 |
| SYT6 | Synaptotagmin 6 | rs11102731 | C > T | 0.50 | 0.007888 |
| TBX5 | T-box transcription factor 5 | rs6489964 | T > A, C | 0.26 | 0.00677 |
| TENM2 | Tenaurin transmembrane protein 2 | rs10626330 | AC > -, ACAC | 0.30 | 0.002343 |
| TGM2 | Transflutaminase 2 | rs6014318 | G > A, C, T | 0.21 | 0.004105 |
| THSD4 | Thrombospondin type 1 domain containing 4 | rs12915131 | T > C | 0.10 | 0.006067 |
| THSD7A | Thrombospondin type 1 domain containing 7A | rs10277192 | C > G, T | 0.48 | 0.00177 |
| TLR5 | Toll like receptor 5 | rs141084293 | - > GACAA | - | 0.004434 |
| TLX1NB | TLX1 neighbor | rs2742053 | A > G, T | 0.31 | 0.0023 |
| TMEFF2 | Transmembrane protein with EGF like and two follistatin like domains 2 | rs114204542 | T > G | 0.18 | 0.002777 |
| TMEM132E | Transmembrane protein 132E | rs1860276 | A > C, G, T | 0.25 | 0.007731 |
| TOB1 | Transducer of ERBB2, 1 | rs8065514 | A > G, T | 0.09 | 0.006067 |
| TOGARAM2 | TOG array regulator of axonemal microtubules 2 | rs6753936 | T > A, C | 0.20 | 0.004678 |
| TRIO | Trio Rho guanine nucleotide exchange factor | rs376318291 | - | - | 0.001565 |
| TSPAN18 | Tetraspanin 18 | rs7104896 | G > A, C | 0.50 | 0.006102 |
| TXNIP | Thioredoxin interacting protein | rs78848289 | A > G, T | 0.12 | 0.007731 |
|  |  | rs9245 | T > C, G | 0.11 | 0.007731 |
|  |  | rs79906727 | G > A | 0.08 | 0.005134 |
| USP12 | Ubiquitin specific peptidase 12 | rs4769540 | A > C | 0.44 | 0.004678 |
| USP24 | Ubiquitin specific peptidase 24 | rs34866370 | A > - | 0.32 | 0.005134 |
| ZAR1L | Zygote arrest 1 like | rs206109 | G > C | 0.45 | 0.006415 |
| ZBTB40 | Zinc finger and BTB domain containing 40 | rs113334 | G > A, C, T | 0.42 | 0.007568 |
| ZNF71 | Zinc finger protein 71 | rs12459820 | G > C, T | 0.40 | 0.002598 |
| ZNF799 | Zinc finger protein 799 | rs3979153 | G > C | 0.05 | 0.003389 |
| ZSCAN5A | Zinc finger and SCAN domain containing 5A | rs10419849 | C > A | 0.12 | 0.002777 |

MAF = Minor allele frequency in references (1000Genomes)

SNPs, single nucleotide polymorphisms; PTB, preterm birth
