# Supplementary material for: A genome-wide and candidate gene association study of preterm birth in Korean pregnant women
Source: PLoS One. 2023 Nov 29;18(11):e0294948. doi: 10.1371/journal.pone.0294948 (PMC10686439; doi:10.1371/journal.pone.0294948)
Supplement: S2 Table — (DOCX) [file pone.0294948.s002.docx]

**S2 Table.** **Reference pathways related to two clusters according to protein-protein interaction**

| ***Cluster 1:* TBX5, RYR2, AKAP6, RPS6KA2** | | | | | |
| --- | --- | --- | --- | --- | --- |
|  | GO-term | Description | Related gene symbol | Strength | FDR |
| Biological process | GO:0003214 | Cardiac left ventricle morphogenesis | GSK3A, HAND1, FOXF1, SFRP2, NOTCH1, TBX5, NPY2R, SMAD4, RBPJ, TGFBR2, RYR2, HEY2, CPE, NPY5R | 2.84 | 0.0483 |
| Molecular function | GO:0034237 | Protein kinase A regulatory subunit binding | SPATC1L, WASF2, AKAP1, GSKIP, PRRC1, AKAP7, AKAP8L, AKAP5, WASF1, ARFGEF2, AKAP14, PRKACB, EZR, ACBD3, RYR2, PJA2, AKAP9, C2orf88, WASF3, PRKACA, AKAP6, AKAP8, ARFGEF1, KCNQ1, AKAP11 | 2.59 | 0.0355 |
| Cellular component | GO:0014701 | Junctional sarcoplasmic reticulum membrane | ASPH, JPH2, RYR2, RYR1, JPH1, JPH3, AKAP6, CASQ2, TRDN, JPH4 | 2.99 | 0.0035 |
|  | GO:0034704 | Calcium channel complex | HSPA2, FKBP1A, CACNA1I, CACNG6, CATSPERB, CASQ2, CACNG4, PKD1, TRPC5, CACNA1C, CACNG8, CALM2, AKAP6, CATSPER3, CACNA1D, PKD1L1, CACHD1, CALM3, CACNG2, CACNB3, CATSPER1, PRKACA, CACNB2, PKD2L1, SMDT1, STAC3, PDE4B, CACNA1H, PDE4D, CACNG1, CACNG3, CATPERG, CATSPER4, CACNA2D1, CALM1, ATP2A1, CACNA1G, RYR1, CACNA1A, MICU1, CACNA1S, C1orf101, RYR2, CACNA1E, CACNA1B, MCU, CACNA1F, ASPH, TRPC4, FKBP1B, CATSPERD, CATSPER2, MICU2, CACNA2D4, RYR3, CACNG7, CACNA2D2, CACNA2D3, CACNB4, TEX40, PPP2R4, ORAI1, CACNB1, CCDC109B | 2.18 | 0.0379 |
| ***Cluster 2:* IFNA21, IL21, LIFR, LRP1B, NTRK2, FHIT, CSMD1, GPM6A** | | | | | |
|  | Pathway | Description | Related gene symbol | Strength | FDR |
| KEGG pathways | Hsa04630 | JAK-STAT signaling pathway | IFNA21, IL21, LIFR, THPO, OSM, IL2RB, SOS2, EPOR, PIK3R2, CSF3, IL2, CCND1, etc. (total 160) | 1.66 | 0.0103 |
|  | Hsa04060 | Cytokine-cytokine receptor interaction | IFNA21, IL21, LIFR, CX3CL1, CD4, TNFRSF17, RELT, TNFRSF1A, NGFR, THPO, OSM, IL2RB, CCL22, CCL17, etc. (total 282) | 1.42 | 0.0272 |

FDR = False discovery rate
