# Supplementary material for: A genome-wide and candidate gene association study of preterm birth in Korean pregnant women
Source: PLoS One. 2023 Nov 29;18(11):e0294948. doi: 10.1371/journal.pone.0294948 (PMC10686439; doi:10.1371/journal.pone.0294948)
Supplement: S3 Table — (DOCX) [file pone.0294948.s003.docx]

**S3 Table.** **The 25 SNPs and their respective probe sequences of the Kompetitive allele-specific polymerase chain reaction (KASP) genotyping platforms**

| Gene name | SNP ID | Sequence |
| --- | --- | --- |
| TBX5 | rs6489964 | TGGAGTCTCGCTCCGTCACCCAGGCTGGTGTGCAGTGGCACGATGTCGGCTCACTGCAACCTCTGCCTCCCAGGTTCAAGTGATTCTTGTGCCTCAGCCT[C/T]TCTAGTACCTGGAATTACAGGTGCGCACCACCATACCTGGTTAATTTTTGTATATTTAGTAGAGATGGGTTTTCGCCATGTTGGCCAGGCTGGTCTCGAA |
| RYR2 | rs2485579 | CCTGGGAATCTGGGTTGTGAGGCTACACCTGTTGGAGGGATCGGAGGGGCCCAGTATATGATACTCCCCTTTCCTGCCCAGCTTCTGAATGAATTGAGAC[A/G]AAAACAATGACTAGGACAAATGAGAGGTCAAAATGTCCTCATAGAGGCTTGGCTGGAGGATGCTGCGTTAAGTCAGCTAGCCCAGTAGGCCTGCTAATTG |
| AKAP6 | rs1950695 | ATTTTTTCTGTTGTGTTTCTGATTGTTGACTCTTTACATTTAGTTTTATCCTTTTAAAAATAAAATATTATTACATCAGTAGTTATTATTCTTTCAGGTT[C/T]CATGATGTTTGCAAACCTCTTAGTTGGCAGTCTTCTATATGTGCTGTTAACATTCTGCATACCTCCTCTTCCACATCATCAATAAAGATA |
| RPS6KA2 | rs6909289^a^ | - |
| INFA21 | rs2891157 | ATCATGGCCATATTTGCTGCAATTATAAGACAATGACAAAAATTTCCATTCAGCTTTTACTCAGAATCAATAATGCCCATTAAGTTTACTGCTAAAACCA[G/C]TGGGATTCTGGGGATTCAAGTTGCAAGAGCAGCATAATATTATCCTTTGCCTGGCACTATATTAACTATTTATGACCTTATCTCTAATCTGTCCTCTACT |
| LIFR | rs3097235 ^a^ | - |
| IL21 | rs309392 | TTTGACAGGAGGCAGCCCACTGAAAATCCAAGTTCTCTCTTCCTCCCCCAGACCATAATCTTACAGTGCATCACTACTGATGCACTGATATATATATTTT[G/T]CCAAGTGCTAAAGGGCTGGAAATTAAAGTCAATATTTGACTGAAGCCAAGGCCAAAACCGAATATTGCCATTAAAAACTTCTAGGAATCAAGAATCAATA |
| LRP1B | rs6758426 | ACAGACACATTTATTATTAGTAAATAGATAAAAACTCACTGGCAAGTTAAATTCTGATTATGTTAATATATTCATTTACATTCTAGTTATATTGTTTTGT[C/T]GTTCCTTAAATGGAGGAATCATGTGATAAAAGTTTAAGTCCAGTTTCACATGTAGGATTTTAGAAGGGGAATAAATTATACCTCAATAAT |
| NTRK2 | rs531904 | ACAATGATGAACAAGCTGGAGTTGAAGAGGCCAAAGAAGCAGGAGAACTTAAGACTTCCAGGAATTCTCAGAAATATTTGGACGAGCTTTGGACTGTCTC[G/A]TCTCCTCAAATCTCAAGGACTGACATGCGGTTCAGAATTGACATGCCTGAATTATGTAAGAAAACCCCAAAACAAAATTGCACCTACCTTAAAAGCAAGA |
| CSMD1 | rs2189890 | GACATATTCATACTTTTTTATAATACCCCCTAAGACACATACCTACCTCTTAGGTTTTTTGTTTGTTTTGTTTTAATCAGTAGAGCAGAAATATCATGGG[T/C]AATTGAAATCCAAAAGGAACATGAAAAGTAAACCAAATAAAAAGAAAACAGCCACCTCTCTGTAATCCAAATTCCCGGGTCATTGCCGCCGTTTGAAGGC |
|  | rs2627403 | TGGGGACCTTTATTAATTTATTTTATTCAAGTTTTATTTTTTAAATATATACTATCTGCATAACAAAATGTAGAAGGTACAGCAGCCAAGCAGTAAAGGC[T/C]TTTCCTTATACCTTTAACCTCAGTCACCCAATTTACCCTATGAAACTAATTGTCTATTTGTCTATACACACACACACACACACACACACACACAAACACC |
| FHIT | rs2736743 | TGAATTCATTTTCCTGTGGCTATGATTGCCCTGAACCCTGTTCTTTGGGTCTTCAAACCAAAAACACTGTGAGTTTTCTATTGGAGGATGGCCACCCGAA[A/G]TGACATTAATTTTAGCCTGTTCTCACTCTAAAAGTTGTAAAATGGGAAACTCACTCTGGACCGATCTGTGCTTCCAAGTTTTGAATTTCAGCTCCCCTCC |
|  | rs2205351 | TTATTGTATGCAGAGCTTCAATCCTGTTATAGTTTTCTGAAGGGTATTGATTTATTTGTTTTAGCAGGCAATTATTTATACTCAAATTTAAAACTATCCC[T/C]TGGGTAGCAGCTCGAATCTCAGTTCAGTTCTTTTATCTTTGTGATAATTTGTGTCTGCTCCATCAAGAATATACACATGACATATACCTCAGAATTACAT |
|  | rs2205350 | GGTTGTATGATATGCAAGTATTGATGTTATTGTATGCAGAGCTTCAATCCTGTTATAGTTTTCTGAAGGGTATTGATTTATTTGTTTTAGCAGGCAATTA[T/G]TTATACTCAAATTTAAAACTATCCCTTGGGTAGCAGCTCGAATCTCAGTTCAGTTCTTTTATCTTTGTGATAATTTGTGTCTGCTCCATCAAGAATATAC |
|  | rs2205349 | TAATTGCTTTTTGTCTTCAGAATGAGTCACATTTTATTGGGTTTCCATATAGCGTGTAATTTGGGGTTGTATGATATGCAAGTATTGATGTTATTGTATG[C/A]AGAGCTTCAATCCTGTTATAGTTTTCTGAAGGGTATTGATTTATTTGTTTTAGCAGGCAATTATTTATACTCAAATTTAAAACTATCCCTTGGGTAGCAG |
|  | rs2594146 | CTGTTCTCACTCTAAAAGTTGTAAAATGGGAAACTCACTCTGGACCGATCTGTGCTTCCAAGTTTTGAATTTCAGCTCCCCTCCAGAATCTGTCTGCTTA[T/A]TTTTATTCTCTAATGCTTCCAGGTAATTTCTTTTTTCTTTTCTTTTTTCTTTTCTTTTTTTTTTTTTTGTTTTTGGTCTTTTGGTACTTTATAGTTGATA |
|  | rs1018374710 ^a^ | - |
|  | rs2594147 | ACTGTGAGTTTTCTATTGGAGGATGGCCACCCGAAATGACATTAATTTTAGCCTGTTCTCACTCTAAAAGTTGTAAAATGGGAAACTCACTCTGGACCGA[T/C]CTGTGCTTCCAAGTTTTGAATTTCAGCTCCCCTCCAGAATCTGTCTGCTTATTTTTATTCTCTAATGCTTCCAGGTAATTTCTTTTTTCTTTTCTTTTTT |
|  | rs2594145 | GCTTCCAGGTAATTTCTTTTTTCTTTTCTTTTTTCTTTTCTTTTTTTTTTTTTTGTTTTTGGTCTTTTGGTACTTTATAGTTGATATCTGTGAGAATTTC[A/G]GATCCAGTAGAACTTACTTAGCCATATTGGAAATGGAAACAAGTAAAAACATTTAGGAAACATTCCTTTGCATTTCCTAACACCTCTCCTCAAAATACTT |
|  | rs2736741 | TTTTCTTTTTCTTCCCTCTGCAACTTTTGGATCATTTTTTTCTTAATTTAGGATTCTATTTTATCTTTGGATTTTCAGTGGTTTTTCTAGGAATTAAAAC[A/G]TATATTCTTGATCTTTCACAGTCTACTTAGATTTAATTTTATACTATCTCATATAAAATTGGAAAACCTTGCAACTGTATAAATCCATTTGCCACTGCCT |
|  | rs6793486 | TTATTATTATTATTATGGGGTTTTTTTGGCAGGGTCTCACTCTGTCGCCTAGGCTGGAGTGCAATGGCATGATCTTAGCTCACTGCAACCTCCACCTCCC[A/G]GGTTCAAGTGATTCTCCTGCCTCAGCCTCCTGAGTAGCTGGGATTACAGACACCTGCTACCACATCCGGCTAATTTTTGGATTTTTGGTAGAGATGAGGT |
|  | rs2736742 | TGTGTCTGCTCCATCAAGAATATACACATGACATATACCTCAGAATTACATGACGTGATCTCACTTGGAATTTTATCAAGTGCATATTCATCTACAATTC[C/T]TACTTCTTCTTAATATAATGATTTACTATCATGAAATGTTCTCCTTCATCTCTGGTAACTCTTTTTGTCTGAAAGTTTAGTTTGTCTCATATTAATAAAT |
|  | rs2594148 | TTCTCTGGCTACTATCAAGATTCTCTCTTTATTGTTGGTCCTCACCAGTTGGAATATGATGTACCTATGCACAGTTTTCTTCAAATGTATCCCATTTGGG[A/G]TTCACTGCATTTCTTGATCTATGCATTTATGTATTTCACCAAATTTGAAAATTTGGGCCCATTATTTCTTCAAATATTTTGTTCTGTTTCATTCTCTTTC |
|  | rs2594150 | AGAATTCTGGATTAACAGTGTTTTGCTTTTTTTTTTACCCCTCGTTTGTACTTTAAAAACATCATTTCAATTTTTCTGGCCTTTATAAATTCTGATGAGA[C/A]GTCATCGTTCATTTAAAGCATTGTTCCACCAAAGATACCGTGCCATTTTTCTCTGGCTACTATCAAGATTCTCTCTTTATTGTTGGTCCTCACCAGTTGG |
| GPM6A | rs7679873 ^a^ | - |

^a^ Four SNPs were analyzed by Sanger sequencing due to the difficulty of primer preparation.
